# Visual Search Within a Limited Window Area: Scrolling Versus Moving Window

**DOI:** 10.1177/2041669520960739

**Published:** 2020-10-18

**Authors:** Yumiko Fujii, Hiromi Morita

**Affiliations:** Faculty of Library, Information, and Media Science, University of Tsukuba, Ibaraki, Japan

**Keywords:** visual perception, visual search, eye movements, tracking/shifting attention, oculomotor behavior, image scrolling

## Abstract

Every day we perceive pictures on our mobile phones and scroll through images within a limited space. At present, however, visual perception via image scrolling is not well understood. This study investigated the nature of visual perception within a small window frame. It compared visual search efficiency using three modes: scrolling, moving-window, and free-viewing. The item number and stimulus size varied. Results showed variations in search efficiency depending on search mode. The slowest search occurred under the scrolling condition, followed by the moving-window condition, and the fastest search occurred under the no-window condition. For the scrolling condition, the response time increased the least sharply in proportion to item number but most sharply in proportion to the stimulus size compared to the other two conditions. Analysis of the trace of scan revealed frequent pauses interjected with small and fast stimulus shifts for the scrolling condition, but slow and continuous window movements interjected with a few pauses for the moving-window condition. We concluded that searching via scrolling was less efficient than searching via a moving-window, reflecting differences in dynamic properties of participants’ scan.

On a daily basis, humans read texts and view pictures on the small display of a mobile phone. Acquiring information this way differs drastically from viewing experiences in the natural world. For example, when we directly perceive the visual world around us, we move our head and eyes to fixate on objects we are interested in, but when we perceive images on a small window via a mobile phone, our fixation points are limited to the area of the window. Although there are many experimental studies on reading scrolling text (Dyson & Haselgrove, 2001; Harvey et al., 2019; Kolers et al., 1981; Valsecchi et al., 2013), there are few studies on how humans perceive scrolling images (Loomis et al., 1991). The present study aimed to clarify the characteristics of image perception within a small window by scrolling. This information is important to further our understanding of how humans process visual information and interact with electronic visual displays.

Visual perception is the ability to perceive our surroundings, including colors, patterns, and structures, using the light that enters our eyes. The eyes have two visual fields that work together: the central and the peripheral. The central field processes the high spatial resolution needed to recognize shapes and patterns. The peripheral field has relatively low spatial resolution but is necessary for smoothly perceiving visual stimuli (Bertera & Rayner, 2000; Foulsham et al., 2011; Saida & Ikeda, 1979). In one study, Saida and Ikeda (1979) had participants view a picture through a square window around a fixation point that followed their eye movements. The image outside the window was masked. When they shortened the window’s side to less than 11 degrees, or half the size of the picture, the processing rate began to decrease. In another case, Bertera and Rayner replaced the characters and numbers with pluses outside the gaze-contingent moving window when participants searched for a target letter. As they enlarged the window, the search time decreased, but it reached asymptotic levels when the diameter of the window was 5 degrees. These studies suggested that perceiving an image within a limited window area was difficult depending on the window area’s size. Accordingly, perceiving images within a small window by scrolling may be difficult due to its limited area, though there are differences between the gaze-contingent moving window method and scrolling mode. The most salient difference is the form of control, that is, by eye movement versus by finger movement, but there are other differences in terms of perceptual properties.

One difference in the perceptual view point is that the screen moves depending on where the viewer looks in the gaze-contingent window method, while in the scrolling mode, the image, rather than the window, is what moves. When humans see a moving image, they try to pursue the target object and keep the image static on the retina. However, when the eyes fail to keep up with the moving target, the image slips on the retina. The image slipping on the retina cannot be perceived as accurately as a static image (Westheimer & McKee, 1975). Therefore, when viewing an image by scrolling, movement may blur the image depending on the pursuit gain and speed of the movement. The speed at which oculomotor systems can pursue a target is about 30 degrees/s (Westheimer, 1954). Thus, scrolling speed is assumed to be closely related to image perception; if scrolling is faster than 30 degrees/s, the visual system can hardly process the image, and it will only be perceived when it pauses. In contrast, if the scrolling moves slower than 30 degrees/s, the eyes can pursue the image.

Another way that scrolling differs from the gaze-contingent eye movement method is that in scrolling, the perceptual properties from the displayed image are overwritten with different visual information with each scroll. That is to say, when we move image quickly to display the desired section, it masks the previously presented section. On the other hand, when viewing with gaze-contingent moving window, part of the image is displayed at each location. Perceiving continuously overwritten stimuli has been investigated using the Rapid Serial Visual Presentation (RSVP) method (Fabre-Thorpe et al., 2001; Potter, 1976; Potter et al., 2002; Potter et al., 2004; Thunell & Thorpe, 2019). Potter (1976) presented pictures by RSVP with very short stimulus-onset asynchrony. She concluded that to identify each picture in sequential presentation required about 100 ms of presentation time for each, but remembering each picture required more than 300 ms (Potter, 1976; Potter et al., 2002). Accordingly, if one scrolls an image by alternately moving and pausing, it may require at least 100 ms of pause to identify each shot and 300 ms of pause to remember each shot.

On the basis of the aforementioned research, our study recognized that displaying images on a small mobile phone screen and viewing them by scrolling may affect perception in the following ways:

1. Sections, limited in size, are presented in sequence.
2. Participant moves the image to display different sections.
3. Sections in a fixed window are overwritten with each other.
4. Recognizing this, our research questions are as follows:

1. Does viewing an image within a small window degrade perceptual performance? If so, how is performance degraded?
2. Does performance deterioration correlate only with window size? Or does it have something to do with the characteristics of scrolling mode, such as movement of image or overwriting of sections?
3. What are the dynamic properties of scan in the scrolling mode? Are sections perceived during pauses or movements?

To answer these questions, we conducted a visual search experiment that compared scrolling versus a moving window.

The procedure asked participants to search for circles among teardrop shapes. According to Treisman and Gormican (1988), a Q or a C among Os is found in parallel because Q and C have distinguishing features such as a small line segment or a cut. However, the search for O among Qs or among Cs is executed serially because the distractor items have distinguishing features but the target item does not. They termed this phenomenon search asymmetry. Accordingly, we assumed that the circle would be searched for serially among teardrops because the teardrop, while similar, included a point.

We varied the number of items and measured participant search time. In case of the serial search, the slope of search time was calculated by dividing search time by the number of items. The slope reflects the time required to scan one item (Treisman & Gelade, 1980). We also varied the size of the search display. In that case, the slope of search time was calculated by dividing search time by the size of the display. The slope reflects how long it took to scan a unit area. Furthermore, we analyzed the trajectory of the image relative to the window to assess the dynamic properties of scanning, such as pauses and movements.

We compared search performance between window and no-window conditions to assess the reduction of search efficiency caused by the limitation of window size. There were two types of window conditions. One was a scrolling condition where participants moved the image under the mask with a window fixed in the center. The other was a moving-window condition where participants moved the entire window to reveal the image below the mask. The size of the window was same between these two conditions so that the researchers could compare search performance.

We hypothesized the following results. The time required to process an item would not depend on the presentation condition, while the necessity to move an image or window would lengthen the search time since the image or window could not be moved as freely and quickly as the participant’s shift of attention. Therefore, the search time would be longer and the slope of search time as a function of display size would be steeper for the window conditions versus the no-window condition.

As shown in Figure 1, if the sections displayed in the window overlapped each other, the same item might be scanned repeatedly for window conditions. This would occur especially with a high-density search display, which may cause the slope of search time as a function of the number of items to increase. In contrast, when the density was low, sections that did not contain an item might be displayed, which can increase search time for display with small number of items to reduce the slope. Accordingly, the slope of search time as a function of the number of items would be steeper or shallower for the window conditions than the no-window condition depending on the proportion of multiple scans of the same item and the frequency of displaying sections with no items.

**Figure 1.**
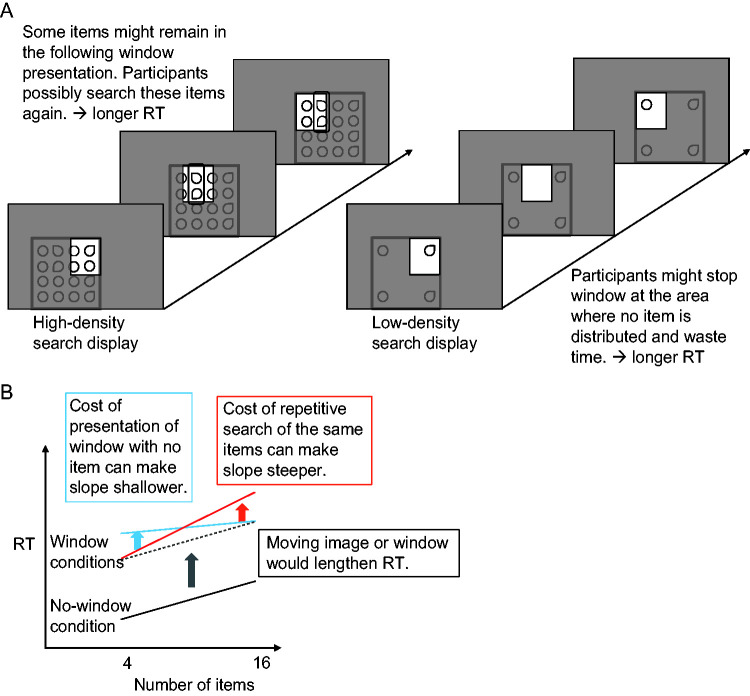
Schematic Illustration of the Relation between the Item Density, the Content of the Window and the Response Time. A: The relation between the item density of search display and the content of the window of three successive presentations. In the case of high-density search display, the two items indicated by the square frame are presented both in the second and third presentation. Participants possibly search these items twice. In the case of low-density search display, no item is included in the second presentation. Participants can waste time presenting the empty section. B: The effect of two types of cost on the slope of response time (RT) as a function of the number of items.

As for the comparison between the two window conditions, the slope of search time as a function of the number of items would not differ between these two modes because the number of items in the window, which depends on the number of items in the search display, are the same between these conditions. However, participants cannot perceive moving images if the scrolling speed is too high. In that case, the search would become intermittent with frequent pauses, which would cause longer search times for the scrolling condition. In addition, the scrolling mode, in which sections of interest are moved into the central window to overwrite the preceding section, would require a longer time to memorize in order to integrate the sequential presentation of sections according to the RSVP studies (Potter, 1976, Potter et al., 2002, Potter et al., 2004). Therefore, the slope of search time as a function of the size of search display would be steeper than that for the moving-window condition. Contrarily, if the search time does not differ between the two window conditions, the scanning method may not affect image perception within a small window.

## Method

The present study was approved by the Ethical Committee of the Faculty of Library, Information, and Media Science at the University of Tsukuba and conducted in accordance with the Code of Ethics and Conduct of the Japanese Psychological Association.

### Participants

The participants included 24 undergraduate and graduate students (11 males, 13 females; age range 21–26 years) with normal or corrected-to-normal vision and normal color vision. Each participant received a full explanation of the experiment and provided written informed consent to participate.

### Apparatus

The visual stimuli were presented on a 23-in. touch-screen liquid crystal display (EIZO Inc., FlexScan T2381W) controlled by a computer (Dell Inc., Dell Precision T1650) in a darkened room. The size of the screen was 511.7 × 288.7 mm (59.1 × 35.5 degrees). The resolution was 1,920 × 1,080 pixels. The display angle was adjusted so that the seated participants could easily operate the screen. We did not use a chin rest because fixating participants’ heads with one would have made it inconvenient for participants to use the touch operation. Consequently, the viewing distance differed among participants. We estimated the viewing distance as 45 cm when calculating the visual angle presented here.

### Stimulus

**Figure 2.**
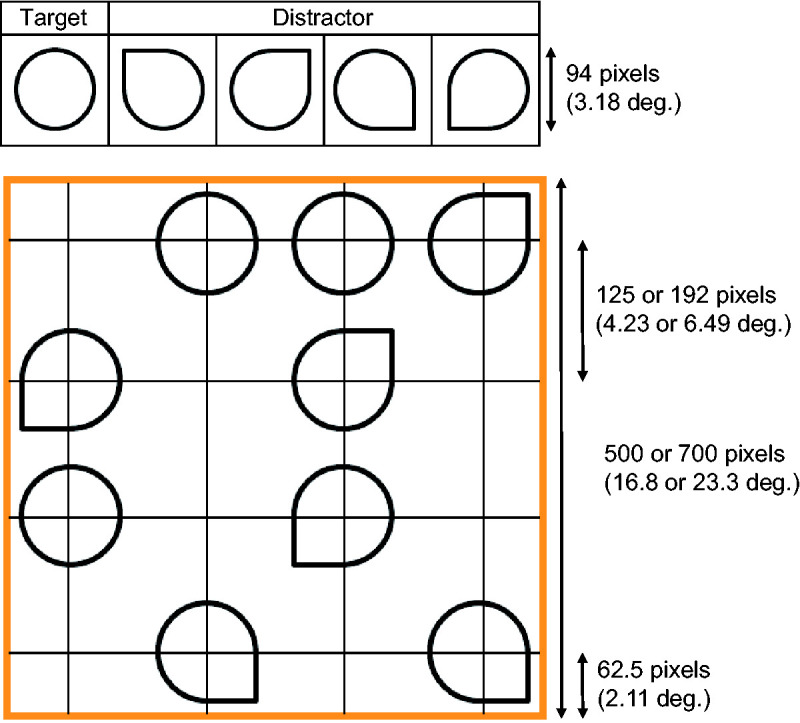
Stimuli. A: Items appearing on the search display. B: An example of the search display with nine items including three targets. *Note*. The grids were not presented in the experiment.

To have participants scan the entire image, we employed circles as targets and teardrops as distractors. There are other types of search displays that are searched for serially, such as the search for rotated T among rotated Ls or the search for the conjunction of visual features. However, searching for rotated T among rotated Ls requires attention to focus on the junction of two line segments. In searching for a conjunction of features such as color and orientation, it was not always required that the attentional spotlight focus on different colored items from the target to reject them. In contrast, to identify teardrops, the attentional spotlight had to focus on the circumference of the item. The search displays were created by arranging the target and distractor items on a white square background—31.2 cd/m^2^, 500 pixels (16.8 degrees) or 700 pixels (23.3 degrees) on each side. The targets were black circular frames 94 pixels (3.18 degrees) in diameter (0.05 cd/m^2^, line width of 5 pixels), and the distractors were teardrop-shaped frames made from the target and a corner of a 94-pixel per side square frame. There were four types of distractors with different orientations (Figure 2A).

A search display comprised 4, 9, or 16 items, including 0, 1, 2, 3, or 4 targets. Each item was placed with its center on one of 16 intersections of a 4 × 4 virtual grid on the background (Figure 2B). Neighboring horizontal lines or vertical lines were separated by 125 pixels when the background was small and 192 pixels when the background was large. Outermost lines were separated from the edges of the background by 62.5 pixels for both background sizes so that participants had to search to the edge of the displays regardless of its size. For the same reason, on the outermost lines, the point of the teardrops, the feature that differentiated the distractor from the target, pointed outward. An orange inner frame for the background (18.0 cd/m^2^, line width of 7 pixels) was presented so participants could easily find the end of the search display.

### Procedure

**Figure 3.**
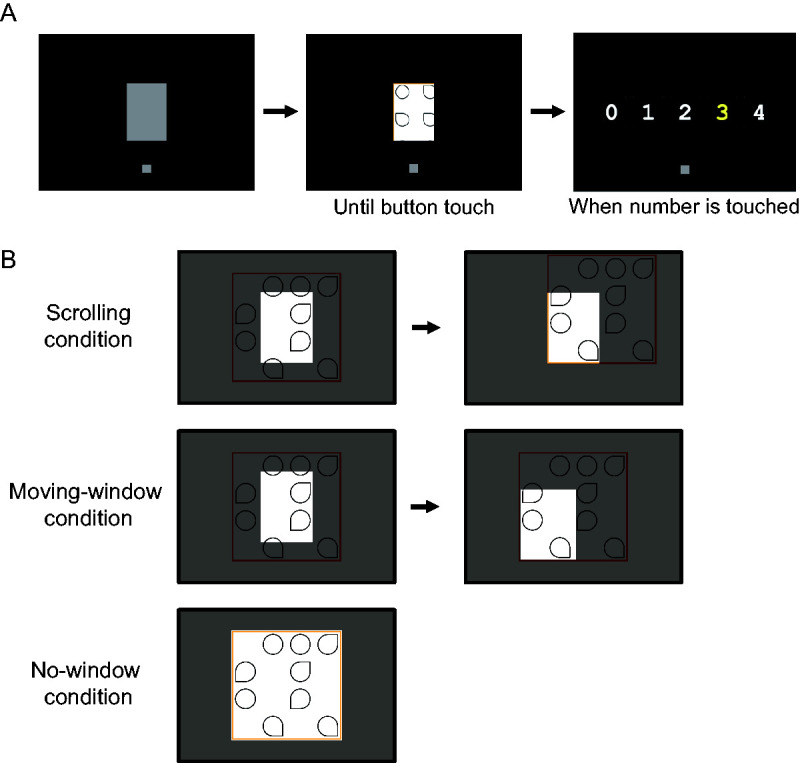
Schematic Illustraton of the Experimental Procedure. A: The sequence of an experimental trial. Participants touched the start-and-stop button to start (left). The search display was then displayed in the window. When they finished the search, they touched the button (center). Then numerals were presented, and participants touched the number of targets they found (right). B: Positional relationship between the search display and the window for three conditions. *Note.* The mask is gray and transparent in this figure, but in the actual trial it was black to mask the search display in the experiment.

The trial started by presenting the start display (Figure 3A). For the scrolling condition and the moving-window condition, a 280 × 400 pixel (9.45 × 11.9 degrees) window filled with gray (10.5 cd/m^2^) was presented in the center of the display. A square window may have been simpler, but we used a rectangular one to simulate a 5.0-in. smartphone because the rectangular window enables participants to scroll in a manner that better replicates a smartphone screen. The start-and-stop button, a gray square button of 60 × 60 pixels, was presented below the window. The center-to-center distance between the window and the button was 390 pixels. When participants touched the button, the search display appeared in the window. As shown in Figure 3B, for the scrolling condition, participants moved the display by touching and sliding their index finger in the window. By doing so, they could see the desired section (lower left section in the Figure 3B) in the central window. By contrast, for the moving-window condition, the display was fixed at the center of the screen and participants moved the window by touching and sliding their finger within the window to the desired section. The search display/window could be controlled only while participants were touching the window. The display/window followed their index finger as they moved it across the screen. Once the finger went out of the boundaries of the window, the movement of display/window stopped until they touched within the window again. When they reached the end of the image, they could no longer move the image/window in that direction. For the no-window condition, the start display comprised only the start-and-stop button. When participants touched the button, the full search display was presented. The display was refreshed in 60 Hz with no interlace. Combining MATLAB® with Psychotoolbox (Brainard, 1997; Kleiner et al., 2007; Pelli, 1997) enabled the program to perceive the current point of touch, calculate the distance between touches, and substitute the section in the window with a new section, which shifted according to the participant’s touch by vertical synchronizing signal. We estimated the delay from touch to redrawing at up to 50 ms. Since the items were larger than the width of an index finger, they were not totally obscured by the finger.

The participants’ task was to search for the targets and count them. As soon as participants completed the search, they touched the start-and-stop button under the window or search display. Response time (RT) was measured from the presentation of the search display until the start-and-stop button was touched. Then numerals 0, 1, 2, 3, and 4 were presented on the screen where participants were required to touch the number of targets they found (Figure 3A).

### Design

There were three viewing conditions: scrolling, moving-window, and no-window. For each condition, participants performed one set of 30 trials—one trial for each combination of the number of items (4, 9, 16), the number of targets (0 to 4), and the size of the search display (small versus large). Participants completed eight practice trials before the experiment with 2-min breaks between sets. The order of the trials in a set was randomized. The order of the condition was counterbalanced among participants.

## Results

Participants whose RTs averaged more than three standard deviations above or below the average for all participants were excluded. As a result, two participants were excluded; the RTs of the excluded participants were 8.36 s and 7.26 s, while the average of other participants’ RTs and their standard deviation were 4.53 and 0.67, respectively.

The purpose of the experiment was to examine whether and how viewing with a window of limited area deteriorates search performance. In addition, whether and how the search performance differs by the mode of presentation while using the same size of the window was examined.

To have participants scan from corner to corner of the search display, we employed multiple target search tasks instead of present/absent tasks where participants may quit the search when they found the target. The goal was to analyze search efficiency in relation to the dynamic properties of scan, such as the number of pauses in the scan process. However, participants who realized there were no more than four targets appeared to quit the search after finding four targets, often before scanning the entire display. Therefore, we excluded the 4-target trials from the analyses.

**Figure 4.**
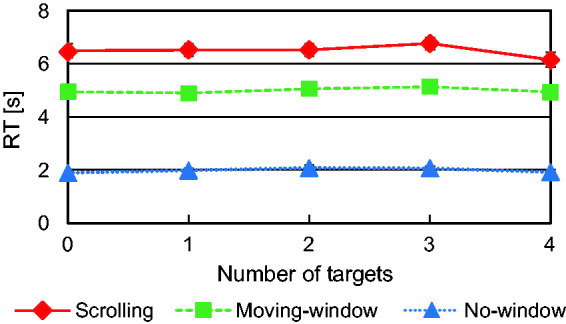
Response Time (RT) as a Function of the Number of Targets.

In fact, the relationship between RT and the number of targets indicated a drop in RT in 4-target trials as shown in Figure 4. We conducted two-way repeated measures analysis of variance (ANOVA) on RT with the presentation condition and the number of targets as factors. The main effects of condition and number of targets were significant—*F*(2, 52) = 260, *p* < .001 and *F*(4, 84) = 4.17, *p* < .005—but their interaction was not—*F*(8, 168) = 1.21, *ns*. Multiple comparisons (Boneferroni corrections were applied for this and other multiple comparisons in the present study) revealed a significant difference between the 4-target and the 3-target trials (*p* < .05). RT for 4-target trials was shorter than that for 3-target trials, which suggests that the participants quit searching before scanning the entire search display in some of 4-target trials. Therefore, we excluded the 4-target trials in the following analyses.

The average error rate was 1.41% (*SD* 2.04%; the highest error rate among participants was 7.8%). The errors in which participants reported fewer than the correct number of targets were considered misses. The rate of these errors was 1.1%. In contrast, when participants reported a number larger than the correct answer, it was considered a false alarm. The rate of those errors was 0.30%.

### Search Time

**Figure 5.**
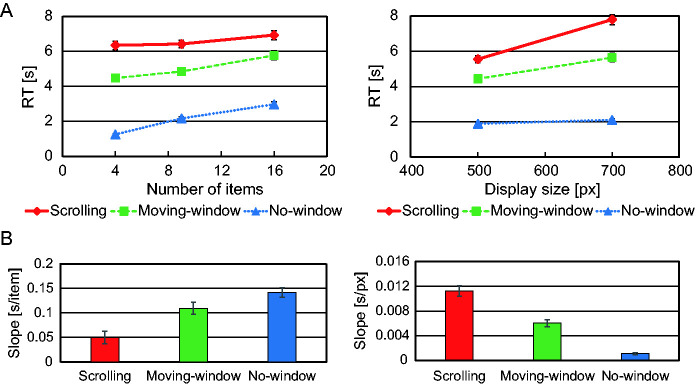
Time Required for the Search. A: Response time (RT) as a function of the number of items (left) and as a function of the display size (right). B: The slope of the response time as a function of the number of items (left) and as a function of the display size (right).

RTs of correct trials with search display with 0, 1, 2, or 3 targets were log-transformed and averaged for each condition combination, number of items, and size of the search display for each participant and then reverse log-transformed. Figure 5A represents the average RT for all participants as a function of the number of items and as a function of the display size.

A three-way repeated measures ANOVA was conducted on RT with three factors: condition, number of items, and size of the search display; it revealed main effects for condition, number of items, and size—*F*(2, 42) = 243, *p* < .001; *F*(2, 42) = 102, *p* < .001; and *F*(1, 21) = 214, *p* < .001. There was no three-way interaction, *F*(4, 84) = 1.83, *p* = .130. For the main effect of condition, multiple comparisons between conditions were significant between each pair of conditions (*p* < .001), showing response time (RT) was longest for the scrolling condition, next longest for the moving-window condition, and shortest for the no-window condition. There was a two-way interaction between condition and number of items, *F*(4, 84) = 20.1, *p* < .001. A post-hoc analysis showed a simple main effect for number of items under each condition (*p* < .01 for the scrolling condition, *p* < .001 for other conditions) as well as a simple main effect of condition for each number of items (*p* < .001). There was also a two-way interaction between condition and display size, *F*(2, 42) = 77.0, *p* < .001. The post-hoc analysis showed a simple main effect for size under each condition (*p* < .001) and a simple main effect of condition for both sizes (*p* < .001).

We calculated the RT slopes as a function of number of items and as a function of display size (Figure 5B). A one-way repeated measure ANOVA on RT slope as a function of the number of items with condition as a factor showed a significant main effect, *F*(2, 42) = 25.0, *p* < .001. Multiple comparisons revealed significant differences between the scrolling condition and the other two conditions (*p* < .005 between the scrolling condition and the moving window condition, *p* < .001 between the scrolling condition and the no-window condition), but no significant difference between the moving-window condition and the no-window condition (*p* = .103). A one-way repeated measure ANOVA on RT slope as a function of display size also revealed a significant main effect, *F*(2, 42) = 82.5, *p* < 0.001. Multiple comparisons showed a significant difference between each pair of conditions (*p* < .001). In short, the RT slope as a function of number of items was shallower for the scrolling condition than for the other two conditions, while the RT slope as a function of display size was steeper for the scrolling condition, less steep for the moving-window condition, and shallowest for the no-window condition.

In summary, RT was longest for the scrolling condition, second longest for the moving-window condition, and shortest for the no-window condition. Moreover, the search time slopes as both a function of the number of items and a function of the display size were different between the two types of window conditions.

### Categorizing the Trace Into Movement and Pause

To investigate the dynamic properties of participants’ scan, we analyzed the coordinates of the central position of the window in relation to the search display for the scrolling and moving-window condition. The sampling rate was 60 Hz, but no sample was recorded when participants took their finger off the display so that neither the stimulus nor the window moved.

We categorized each period between consecutive samples into Movement or Pause. If the distance between the central window position of the *n*th and *n* + 1st samples exceeded 4.67 pixels (e.g., the speed of the search display or the window exceeded 280 pixels per second; 9.45 degrees/s), the period was categorized as Movement. In addition, if the *n*th to *n* + 1st period was categorized as Movement based on the shift of more than 4.67 pixels, the *n* – 1st to *n*th and/or *n* + 1st to *n* + 2nd periods were also categorized as Movement unless the shift in these periods was equal to zero pixels. In this case, the *n* – 2nd to *n* – 1st and/or *n* + 2nd to *n* + 3rd periods were also categorized as Movement unless the shift was equal to zero pixels. In other words, we considered the accelerating and decelerating periods in a range of three samples before and after the periods of high speed to be Movement. Any period sandwiched between two periods where the search display or window shifted more than 4.67 pixels was also categorized as Movement. The remaining periods were categorized as Pause.

Next, we calculated the total distance for each series of Movement periods. A Movement series where the total distance was less than 70 pixels (a quarter of the window width) were re-categorized as Pauses since such a small shift was assumed to reflect an adjustment to perceive an item as a whole rather than examine something new in the window.

Similarly, we calculated the total time for each series of Pause periods and recategorized the series where the total time was less than 200 ms as Movement. This is because we thought that participants would need at least 200 ms to process visual information during a pause based on the knowledge that humans generally fixate their eyes at more than 200 ms to process visual information. Therefore, movements where participants stopped for more than 200 ms were labeled Pauses in this study. Furthermore, we labeled multiple consecutive Movements as a movement and multiple consecutive Pauses as a pause.

**Figure 6.**
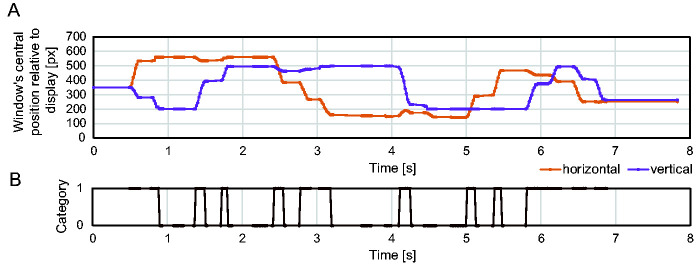
Categorizing the Trace into Movement and Pause. A: Horizontal and vertical coordinates of the window’s central position relative to the upper left of the search display. B: Categorization of scrolling or window movement. *Note.* Zero stands for a pause phase and 1 stands for a movement phase.

**Figure 7.**
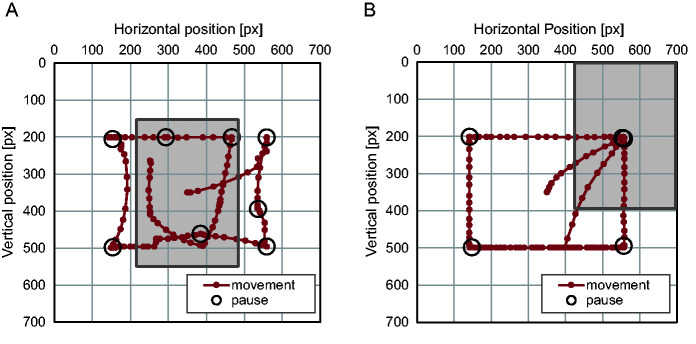
Trace of the Window’s Central Position in Relation to the Search Display. A: The trace for the scrolling condition. B: The trace for the moving-window condition with the same participant. *Note.* The gray rectangle represents the size of the window.

Figure 6 shows the horizontal and vertical coordinates of the window’s central position relative to the search display and their categorization for the scrolling condition. Figure 7 represents the search path for the scrolling condition and the moving-window condition from the same participant. These search paths suggested the search display was scrolled with frequent pauses under the scrolling condition, but for the moving-window condition, the window moved systematically with four pauses at each corner.

### Analysis of the Trace

#### The Number of Pauses

**Figure 8.**
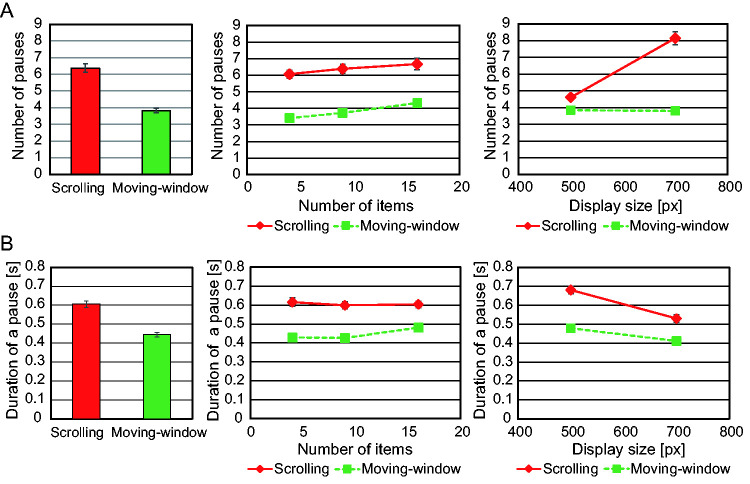
Dynamic Properties of Scan. A: The number of pauses for scrolling and moving-window conditions (left); the number of pauses as a function of number of items (center) and as a function of display size (right). B: Average pause duration for the scrolling and moving-window conditions (left); average duration of a pause as a function of number of items (center) and as a function of display size (right).

Figure 8A shows the average number of pauses per trial. We conducted a three-way repeated measures ANOVA on the number of pauses with condition, number of items, and size of the search display as factors. There were main effects for condition, number, and size—*F*(1, 21) = 129, *p* < .001; *F*(2, 42) = 17.0, *p* < .001; and *F*(1, 21) = 96.2, *p* < .001—but there was no three-way interaction—*F*(2, 42) = 0.007, *p* = .993. There was an interaction between condition and size (F (1, 21) = 132, p < .001); the post-hoc analysis revealed a simple main effect of size for the scrolling condition, *F*(1, 21) = 131, *p* < .001, but no simple main effect of size for the moving-window condition, *F*(1, 21) = 0.134, *p* = .718. The simple main effect of condition was found for both sizes: *F*(1, 21) = 30.8, *p* < .001 for small size and *F*(1, 21) = 144, *p* < .001 for large size. There was no interaction between condition and number of items, nor between size and number of items: *F*(2, 42) = 1.71, *p* = .192 and *F*(2, 42) = 0.197, *p* = .822. In summary, participants paused more frequently under the scrolling condition than under the moving-window condition. The number of pauses increased as the number of items increased for both conditions and as the display size increased for the scrolling condition but not for the moving-window condition.

#### The Average Pause Duration

Figure 8B shows the average pause duration. A three-way repeated measures ANOVA showed a main effect of condition, number of items, and size of the display—*F*(1, 21) = 101, *p* < .001; *F*(2, 42) = 5.11, *p* < .05; *F*(1, 21) = 96.7, *p* < .001—but there was no three-way interaction—*F*(2, 42) = 1.38, *p* = .264. There was an interaction between condition and number of items as well as between condition and stimulus size—*F*(2, 42) = 6.78, *p* < .005 and *F*(1, 21) = 13.5, *p* < .005—but there was no interaction between number and size, *F*(2, 42) = 2.84, *p* = .07. Post-hoc analysis revealed a simple main effect for condition on each number of items—*F*(1, 21) = 54.8, *p* < .001; *F*(1, 21) = 138, *p* < .001; and *F*(1, 21) = 62.2, *p* < .001 for 4, 9, 16 items, respectively—with a simple main effect for number of items for the moving-window condition, *F*(2, 20) = 9.58, *p* < .005, but not for the scrolling condition, *F*(2, 20) = 0.827, *p* = .452. Post-hoc analysis also showed a simple main effect for condition on both sizes—*F*(1, 21) = 143, *p* < .001 for small size and *F*(1,21) = 29.5, *p* < .001 for large size—as well as a simple main effect for size on both conditions—*F*(1, 21) = 82.8, *p* < .001 for the scrolling condition and *F*(1, 21) = 19.9, *p* < .001 for the moving-window condition. In summary, participants spent more time pausing under the scrolling condition than under the moving-window condition. The pause duration increased as the number of items increased under the moving-window condition but not under the scrolling condition. Pause duration decreased as the size of the display increased for both conditions.

#### The Average Speed and Distance of a Movement

**Figure 9.**
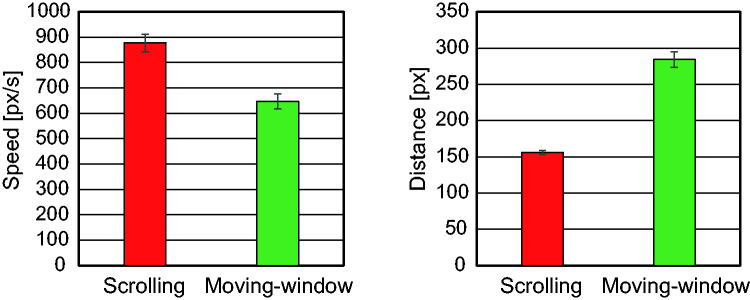
Average Speed (Left) and Distance (Right) of a Movement for the Scrolling Condition and for the Moving-Window Condition.

Figure 9 shows the average speed and distance of a movement of the search display or the window. A one-way repeated measures ANOVA on speed with condition as a factor revealed the main effect of condition, *F*(1, 21) = 145, *p* < .001, which showed that the search display was scrolled faster than the window was moved. A one-way repeated measures ANOVA on distance also revealed the main effect of condition, *F*(1, 21) = 141, *p* < .001), which showed the window moved a longer distance with the moving-window movement versus the scrolled movement. These differences in the dynamic properties of scan might be involved in the difference in search performance.

## Discussion

### Search Time

The results showed that, given equal window size, scrolling was more difficult than searching by moving the window. This suggests that search performance with a limited window area depends on the mode of presentation. To our knowledge, this has never been shown before. We infer that the inferior performance with scrolling mode can be attributed to the movement of the search display and/or overwriting sections with each other.

As we predicted, RT was longest for the scrolling condition, second longest for the moving-window condition, and shortest for the no-window condition, which showed that search performance decreased when the visible area was limited to the window. Bertera and Rayner (2000) showed that search performance decreased as the size of the eye-contingent window shrank. Our findings were consistent with those results because the limitation of the visible window area affected search performance. However, our results varied in that a window size of greater than 5 degrees (here 9.45 × 11.9 degrees) affected the performance. This contrast may relate to variations in target shape and size or to the items located outside the viewer window. Bertera and Rayner presented letters and numbers smaller than 0.3 degrees, but we presented larger shapes: circles and teardrops with a 3.18 degrees diameter. In addition, in their study, the items outside of the window were replaced with pluses, but our study completely blacked out the area outside of the window. Moreover, this discrepancy could result from variation in how participants controlled the window, that is, by gaze or by finger.

Among the three conditions, the scrolling condition yielded the shallowest search time slope as a function of number of items but the steepest search time slope as a function of display size. This indicated that for the scrolling condition, items in the window were searched almost in parallel, while the search display was scanned serially by the window. In contrast, under the moving-window condition, the search time slope as a function of the number of items was as steep as that of the no-window condition, and search time slope as a function of the size of the search display was shallower than that under the scrolling condition, though window size was held constant between scrolling and moving-window conditions. This indicated that the performance in the moving-window search fell between that in scrolling and that in no-window searches. Next, we discuss slope differences between the window mode types by considering the dynamic properties of scan.

### Trace of Scan

For the scrolling condition, the scan paused more frequently, and the average pause duration was longer compared with the moving-window condition, which might be reflected in the response time difference between conditions.

The number of pauses increased from five to eight as the search display enlarged for the scrolling condition. By contrast, for the moving-window condition, the window paused only four times, in most cases at the four corners of the search display, irrespective of the stimulus size. Since a 280 × 400 pixel window can scan all over a display of 500 × 500 pixels with four pauses and a display of 700 × 700 pixels with six pauses, the window contents would have overlapped between successive pauses under the scrolling condition, suggesting some items would have appeared repeatedly in separate but overlapping sections. In contrast, under the moving-window condition, there was an area not displayed during any pauses when the search display was large, suggesting some parts of the display were searched while the window was moving.

Regarding whether scrolling or window movement was slow enough for visual processing, the average scrolling speed was 877 pixels/s (29.0 degrees/s) and the window movement speed was 646 pixels/s (21.6 degrees/s). Since the visual system cannot pursue a target moving above 30 degrees/s (Westheimer, 1954), this confirms that participants searched mostly when they stopped scrolling. It has been reported that the maximum velocity of smooth pursuit is improved when sinusoidal movements of a target is tracked with the eyes and hand than when it is tracked with the eyes alone (Gauthier et al., 1988; Koken & Erkelens, 1992; Niehorster et al., 2015). However, when the target moved randomly, relatively small or no difference has been found between these two tracking conditions. Since participants moved the image intermittently step by step in the scrolling condition in the present experiment, we do not assume that their eyes could smoothly pursuit a target with velocity much higher than 30 degrees/s. In contrast, during the moving-window condition, although participants could pursue the window, they would not do so but, instead, fixate an item in the static display while moving the window. Then, they would follow the window by making saccades. The average scrolling distance was 156 pixels (5.27 degrees), around half of the window width, and the window movement distance was 284 pixels (9.58 degrees), close to the window width.

These dynamic properties of scan showed that participants quickly shifted the search display by short steps and frequently paused to search in the scrolling mode, while they made slow and long window movements during which they continued to search in the moving-window mode. This is confirmed by pause duration, which was shorter for the moving-window condition than for the scrolling condition.

The pause duration increased as the number of items increased for the moving-window condition. This was because item density within the window frame increased along with the number of items. However, the pause duration did not depend on the number of items for the scrolling condition. This may relate to the frequency of pauses, which increased as the number of items increased. When participants moved the search display, new items were introduced in the window. If they moved the search display by short steps, only a small number of items appeared in the window. Since it is not likely that the participants searched the same items, according to the low false alarm rate, it may be that by moving the display in short steps, participants only searched a few new items in the window at every pause. For example, the highest density display (a small search display with 16 items) was searched with 4.9 pauses, and it was calculated that 3.3 new items appeared in the window per pause. If we assume that the small number of items could be processed nearly in parallel, then the duration of pauses did not depend upon the number of items.

It might be worthwhile to compare the dynamic properties of scan with the eye movement results of Zelinsky and Sheinberg (1997). In Experiment 1, they employed asymmetrical serial and parallel search tasks using O- and Q-like stimuli and found that the number of saccades highly correlated with RT, while the latency of saccades did not. They arranged 5 or 17 items at 3, 4, 5, or 6 degrees from the center of the display. The diameter of O was 0.67 degrees and the length of a line segment of Q was 0.67 degrees. As a result, RT showed the search asymmetry and a 1:2 ratio at an increasing rate for target-present trials compared to target-absent trials with a serial search. The number of saccades showed the same pattern as that of RT, but the initial saccade latency did not. If we focus on the results of the serial search, the number of saccades did not increase, but the latency of the initial saccade increased significantly with an increase in the number of items. While not a direct comparison, if we contrast the number of saccades in their study to number of pauses in ours, the pauses increased as the items increased, while the number of saccades did not increase significantly. Nevertheless, an increasing tendency was seen in the graph for target absent trials. The pause duration did not increase with the increase in the number of items, while initial saccade latency increased in Zelinsky and Sheinberg (1997), though they reported that subsequent saccade latency had no relevance to experimental search manipulations.

For the scrolling condition, when the number of items increased, pause duration did not increase, but the number of pauses did, which resulted in a longer search time with the high-number-of-items condition. On the other hand, there were section presentations with no new items with the low-number-of-items condition. For example, the number of pauses was six for trials with four items, which meant that for at least two pauses, no new items were presented. These useless pauses could have lengthened search time, especially when a small number of items were displayed. It might be that these two contradictory factors affected the slope of RT as a function of the number of items for the scrolling condition.

For the moving-window condition, both the pause duration and the number of pauses increased as item number increased. In addition, the large distance of movement and the ability to search during the window movement ensured no useless pauses where no new items were presented. The ability to search during window movement seemed to reduce the limitation created by the window, which resulted in a rather small reduction in performance for the moving-window mode compared with the scrolling mode.

The average pause duration under the scrolling condition was 606 ms, which was longer than the presentation time required to identify or recognize the RSVP stimulus reported by Potter and colleagues (Potter, 1976; Potter et al., 2002). We cannot directly compare the results of their experiments to ours since the stimuli and tasks differed between these experiments, but it is plausible that the additional time to determine the next pause position increased the scrolling pause.

Finally, the scan traces were quite regular when compared with the gaze shift measured by eye movement studies (e.g., Engel, 1977; Gilchrist & Harvey, 2000; Hooge & Erkelens, 1996). Compared with the eye movement control, which has been suggested to draw on currently available visual input and on several cognitive systems (Henderson, 2003), scrolling or moving a window is not as free of a movement and is completely under conscious control. Therefore, investigating scan traces might have some implications for studies on conscious control of eye movement and attention.

von Mühlenen et al. (2003) conducted visual search experiments with restricted windows where participants could see only one quadrant of the dynamic search display. In the dynamic search display, items were relocated randomly every 100 ms or so (Dickinson & Zelinsky, 2013; Horowitz & Wolfe, 1998). von Mühlenen et al. (2003) found that dynamic search with aperture was as efficient as when the full display was visible, suggesting that a sit-and-wait strategy was used in searching dynamic displays. Items were relocated randomly in their search stimuli, but in our study, they were shifted by participants’ finger scrolling.

## Conclusion

We showed that searching with a window smaller than the entire display decreased search performance, with a lower performance for scrolling than moving-window searches. We suggested the difference in search performance is related to differences in the dynamic properties of scan between the two modes. In the scrolling mode, the search was conducted during pauses interjected with small stimulus shifts, while in the moving-window mode, the search was also conducted throughout slow and long window movements. As a result, the same-sized display area elicited a different performance depending on the viewing mode, which indicated the importance of how images were presented within a limited window frame. We believe that this study is important as the first step to deepen our understanding of how the visual system perceives scrolled images and to improve how images are displayed within a limited space.
